# Sensor Cell Network for Pressure, Temperature and Position Detection on Wheelchair Users

**DOI:** 10.3390/ijerph19042195

**Published:** 2022-02-15

**Authors:** Cátia Tavares, Daniela Real, Maria de Fátima Domingues, Nélia Alberto, Hugo Silva, Paulo Antunes

**Affiliations:** 1 Department of Physics & I3N, University of Aveiro, Campus Universitário de Santiago, 3810-193 Aveiro, Portugal; danielafreal@ua.pt (D.R.); pantunes@ua.pt (P.A.); 2 Instituto de Telecomunicações and University of Aveiro, Campus Universitário de Santiago, 3810-193 Aveiro, Portugal; nelia@ua.pt; 3 Instituto de Telecomunicações, Instituto Superior Técnico, University of Lisbon, 1049-001 Lisbon, Portugal; hsilva@lx.it.pt; 4 PLUX—Wireless Biosignals, S.A, 1050-059 Lisboa, Portugal

**Keywords:** wheelchair user, fiber Bragg grating, pressure and temperature sensors, pressure ulcers, position detection

## Abstract

This work proposes an optical sensing network to monitor pressure and temperature in specific areas of a wheelchair to prevent pressure ulcers and to monitor the position of the wheelchair user by analyzing its pressure distribution. The sensing network is composed of six optical fiber Bragg grating (FBG)-based sensor cells. Each sensor cell is built from a polylactic acid (PLA) base and has two FBGs, one embedded in epoxy resin to monitor pressure variations (FBG_P_) and another without resin to monitor temperature (FBG_T_). Once produced, all sensor cells were experimentally characterized for pressure and temperature variations, resulting in an average pressure sensitivity of 81 ± 5 pm/kPa (FBG_P_) and −5.0 ± 0.4 pm/kPa (FBG_T_), and an average temperature sensitivity of 25 ± 1 pm/°C (FBG_P_) and 47.7 ± 0.7 pm/°C (FBG_T_). The sensor cells were then placed in six specific areas of a wheelchair (four in the seat area and two in the shoulder blade area) to carry out experimental tests, wherein the response of the sensors to a specific sequence of relief positions was tested. During the execution of the test, the optical signal of all sensors was monitored, in real time, with the pressure and temperature values detected in each zone of the wheelchair. In addition, random position changes were performed in order to evaluate the precision of the proposed sensing network in the identification of such positions.

## 1. Introduction

**Figure 1:**
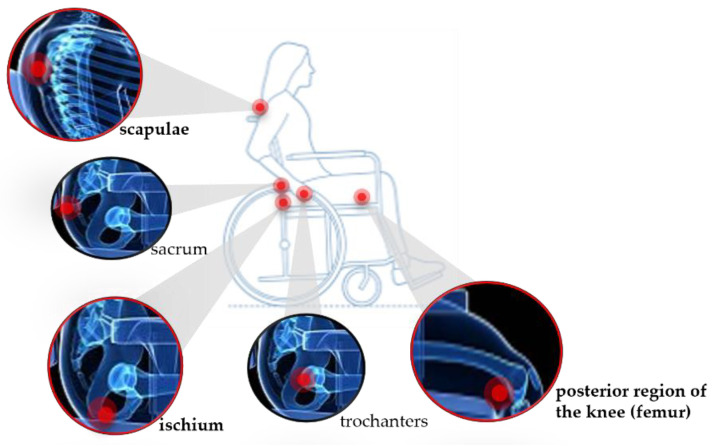
Schematic representation of the most common areas for the development of pressure ulcers, highlighting the areas studied in this work.

The advances in knowledge associated with the development of science and technology, as well as the increase in the average life expectancy of people, has led to a constant search for solutions in the field of health to improve the quality of life of the population. Often, these solutions do not lead to the cure of a pathology, but instead focus on its treatment and/or prevention. In this work, we propose a solution to improve the life quality of people who, for various reasons, have limited mobility and are bound to the daily use of a wheelchair. Due to the prolonged use of such mobility aid, wheelchair users may suffer from different pathologies, with pressure ulcers being one of the most recurrent, which in more severe cases may precede a generalized infection and lead to the death of the patient [1]. A pressure ulcer is a lesion on the skin and/or underlying tissue, resulting from prolonged exposure of a given anatomical region to pressure or pressure combined with torsional forces [2,3]. Additional pressure applied to a specific area of the skin impairs blood flow, resulting in a decrease in oxygen and nutrients to that area (ischemia) when sustained for a long period of time [4]. If this pressure is not relieved, an ulcer will begin to form. Therefore, in wheelchair users, this additional pressure combined with limited mobility, low sensitivity, poor nutrition, ageing of the skin, and increase in temperature and humidity can eventually lead to the development of ulcers [5,6]. The most common areas for the development of this pathology are ischium and sacrum; scapulae; and, in some cases, trochanters and posterior region of the knee, depending on the chair and the position most frequently assumed by the user [7,8] (Figure 1). It is estimated that 95% of wheelchair users develop pressure ulcers during their lifetime [7], which is why it is considered one of the most critical secondary complications for patients with bone marrow damage [9]. Additionally, several studies show that the treatment of pressure ulcers causes high costs for both patients and health services. An article published in 2010 mentions that the treatment of a stage 4 pressure ulcer can cost as much as USD 129,248 per patient [10].

The most common methods to prevent this pathology are movements that relieve pressure and cushions in wheelchairs. Among these, the most cost-effective is the regular repositioning; frequent changes in posture, even sitting, can change the pressure at the skin surface and restore blood flow, improving the tissue health [9]; therefore, individuals with risk of developing pressure ulcers are recommended to change their positions regularly [3,6,9,11]. These repositioning movements include vertical push-ups and side and forward tilts in order to reduce the duration and amount of pressure [6]. Nevertheless, there is no scientific consensus on the frequency with which this type of exercise should be performed nor on the duration of the exercises. To date, the maximum pressures above which their application to specific parts of the body can be considered harmful have not yet been identified [4]. The tolerance of tissues to pressure varies according to the patient, depends on the nature of the tissue and its location, age, hydration, and metabolism. 

There are already some published works with devices designed to monitor pressure in wheelchairs. In 2008, Gassara et al. designed a smart wheelchair with multiple electronic sensors (FlexiForce A201 sensor, optical pulse sensor and SCA11H sensor) for force, temperature, and heart rate monitoring [3]. Later, in 2009, a system was reported for problematic posture detection and notification of the user [8]. In 2012, an article was published on monitoring pressure relief using electronic sensors [4], and in the following year, Chenu’s team published a paper on textile sensors to measure pressure at the interface between the mattress and the ischial area [12]. In 2014 and 2016, other teams reported the pressure monitoring by using electronic sensors to evaluate pressure relief movements [7,13]. More recently, Yang et al. included electronic sensors on wheelchair cushions for temperature and humidity monitoring in ischial tuberosities and thighs, with the aim of preventing pressure ulcers [14]. There is only one paper published in 2019 that reports the use of fiber Bragg grating (FBG)-based sensor network to monitor pressure in different areas of a wheelchair, namely, scapulars, ischiatic, and heels, but this work does not provide temperature compensation at each point of analysis [15].

With this work, we intend to develop a non-electronic system for simultaneous real-time pressure and temperature detection in different areas of wheelchairs in order to help the wheelchair user and their caregivers/medical staff to prevent and control pressure ulcers. This system consists of a network of six sensor-cells, capable of measuring pressure and temperature at each point. This network of sensor-cells has two optical fibers as the main sensing component, each with six FBGs (one fiber for pressure measurement, and the other one for temperature monitoring). This is a system that can be easily adapted to different wheelchairs.

The manuscript is structured as follows: after the introduction provided in Section 1, Section 2 focuses on the sensor-cells sensing principle and design; Section 3 deals with the characterization and calibration of the sensor cells; Section 4 describes the implementation of the sensor cells in the wheelchair and the tests results; Section 5 presents the discussion; and, finally, Section 6 focuses on conclusion and future work perspectives.

## 2. Sensor Cells Sensing Principle and Design

The sensing network, proposed here to monitor pressure and temperature on the wheelchair, is composed by 12 multiplexed FBGs, divided into two optical fibers with six FBGs each.

The Bragg gratings are characterized by a periodic disturbance of the refractive index of the fiber core, normally produced with a UV laser. When an optical fiber with an FBG is illuminated by a broadband light source, only a set of wavelengths that meet the Bragg condition are reflected, with the others being transmitted. The Bragg condition is given by:

(1) $$\lambda_{B}=2n_{eff}\Lambda$$

where *λ_B_* is the reflected Bragg wavelength, *n_eff_* is the effective refractive index of the optical fiber core, and Λ is the grating period [15].

As mentioned previously, the *λ_B_* can be affected by changes in strain (Δl) and/or temperature (Δ*T*). Consequently, the reflected Bragg wavelength varies (Δ*λ_B_*) according to the following equation:

(2) $$\Delta \lambda_{B}=\Delta \lambda_{B,\iota }+\Delta \lambda_{B,T}=2\left(\Lambda \frac{\partial n_{eff}}{\partial l}+n_{eff}\frac{\partial \Lambda }{\partial l}\right)\Delta l+2\left(\Lambda \frac{\partial n_{eff}}{\partial T}+n_{eff}\frac{\partial \Lambda }{\partial T}\right)\Delta T=S_{l}\Delta l+S_{T}\Delta T$$

where the first term is the strain-induced wavelength shift, and the last is the thermal effect on the same parameter, with $S_{l}$ and *S_T_* representing the strain and temperature sensitivity coefficients of the FBG. 

**Figure 2:**
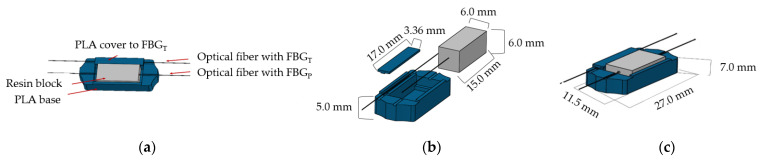
3D design of sensor cell with: (**a**) all components; (**b**) components dimensions; and (**c**) final assembled sensor cell with its dimensions.

The goal of this work was to build a sensing system with six optical sensor cells that monitor the temperature and pressure in specific areas of the seat and backrest of the wheelchair and allow conclusions to be drawn about the position of the user. The six sensor cells have the same design and components, as shown in Figure 2. The base, shown in blue in Figure 2a,b, was printed in polylactic acid (PLA) using a 3D printer (Ultimaker 3D Extended). Each sensor cell contains two FBGs that were inscribed into photosensitive optical fibers (GF1 Thorlabs) using a pulsed Q-switched Nd:YAG laser system operating at the fourth harmonic at 266 nm. The FBGs were recorded using the phase mask technique, employing a laser pump energy of 25 J with a repetition rate of 10 Hz and an exposure time of approximately 1 min. One FBG is responsible for the temperature monitoring (FBG_T_), being protected by a small cover to avoid pressure interferences. In this case the Bragg wavelength change is mostly due to temperature variations. The FBG_P_ is embedded into a block of thermosetting epoxy resin (LiquidLens), with a Young modulus around 6.5 MPa that is in contact with the surface. When pressure is applied to the top of the resin block, it expands, causing strain in the optical fiber that modulates the Bragg wavelength. At the same time, the epoxy resin is also sensitive to temperature variations, changing its shape with temperature fluctuations, and therefore it is expected that there will be variations in the wavelength of the FBG_P_ due to temperature.

The sensor cell design was created with higher PLA walls on the sides of the resin (0.5 mm) and lower walls on the transverse tops so that the resin moves longitudinally and promotes elongation of the optical fiber when it is under pressure. Each sensor cell resin block, shown in shades of gray in Figure 2a,b, is 15 mm long, 6 mm wide, and 6 mm high. Figure 2c depicts the final assembled sensor cell.

Following this design, we produced six sensor cells for pressure and temperature detection on the scapulas (S), ischium (I), and femur (F). Both sides (left and right) were considered, and thus the sensor cells were identified as SL, SR, IL, IR, FL, and FR. As an example, the IR corresponds to the sensor cell positioned on the ischium (I) of the right side (R).

## 3. Sensor Cell Characterization and Calibration

### 3.1. Response Time of the Sensor Cell to Pressure and Temperature Variations

**Figure 3:**
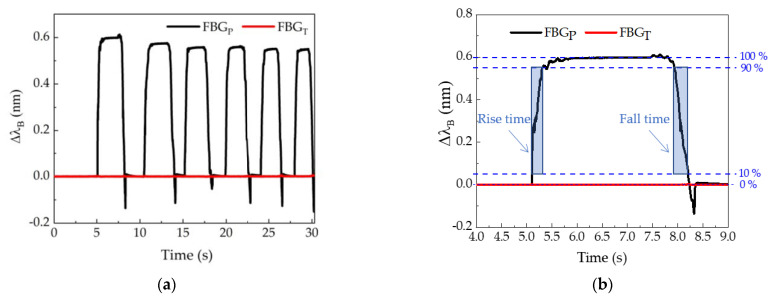
Determination of the response time of the IR sensor cell to pressure variations: (**a**) entire test; (**b**) zoom of first peak to show the methodology used to find the rise and fall times.

The response time of the projected sensor cells to pressure variations was determined by placing and quickly removing a mass of approximately 4 kg on the six sensor cells. The data were collected using an I-Mon USB512 interrogator (Ibsen Photonics) due to its high acquisition frequency (≈1 kHz), with a resolution of 5 pm and wavelength range of 70 nm. According to the results shown in Figure 3a, for the IR sensor cell, as expected, no Bragg wavelength shift was obtained in the case of the FBG_T_ sensor. The determination of the response times of FBG_P_ for the rise and fall of all cycles only considered the analysis of the elapsed time between 10 and 90%, as shown in Figure 3b. An average rise time of 0.30 s and a fall time of 0.38 s were obtained. The difference between the values may have been due to the fact that the resin itself took time during the descent to return to its original position. In any case, both values were quite low for the type of movement to be detected in this work, and therefore the sensor design here proposed was proven to be suitable for the intended application. Since the response time is about 0.5 s, the sm125–500 interrogator (Micron Optics), another interrogation system available in our laboratory, which registers two points per second with a resolution of 1 pm and a wavelength range of 80 nm, is suitable for the sensor calibration, and therefore it was the interrogator used during all tests.

**Figure 4:**
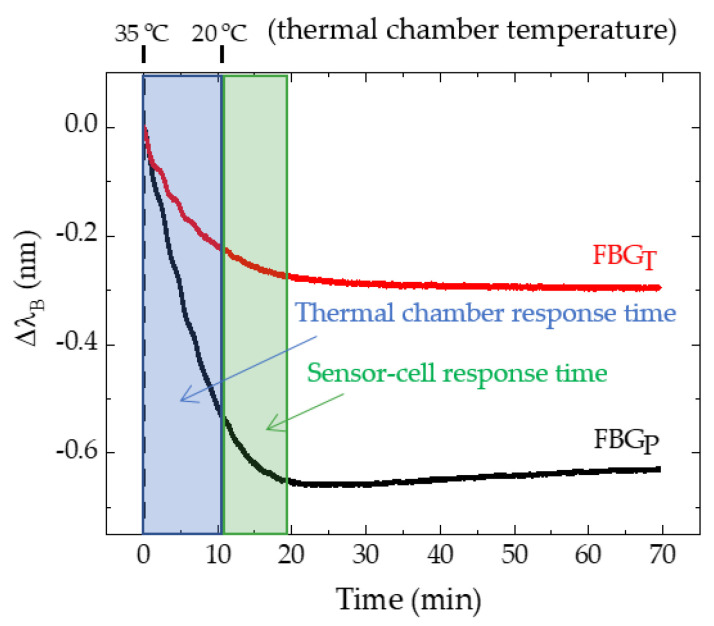
Determination of the response time of the SL sensor cell to 15 °C of temperature variation.

To obtain the response time of the sensors with respect to temperature variations, we placed one of the six sensor cells, in this case the SL, in a climate test chamber (CH340, Angelantoni Industries). The results are presented in Figure 4. 

The Micron Optics sm125–500 interrogator was used to record the Bragg wavelength for FBG_P_ and FBG_T_. At the beginning of the test, at minute 0.0, the thermal chamber displayed 35.0 °C, having been programmed to 20.0 °C at that time. The desired temperature was reached 10.6 min after the start of the test. As shown in Figure 4, 20.0 min after the thermal chamber was turned on, the temperature of the FBGP and FBGT was considered stabilized.

### 3.2. Calibration to Pressure and Temperature

**Figure 5:**
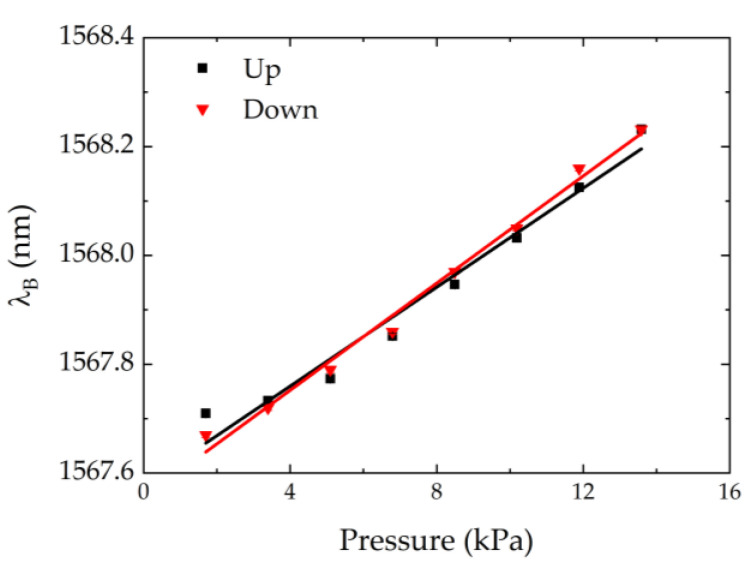
FR sensor cell calibration test to pressure variations. The symbols correspond to experimental data, and the lines to the linear fits.

The pressure calibration test was performed for all sensor cells, ranging from 0 to 15 kPa (using several loads), at a controlled temperature. Results of the calibration test for the FR sensor cell are shown in Figure 5. Since the sensor cell response was also tested for the decrease of the pressure load, the hysteresis of the sensors was evaluated as well. 

For all sensor cells, these tests resulted in a linear dependence of the Bragg wavelength change with the applied pressure. The sensitivity coefficient of each sensor cell was given by the average of the slopes of the linear fits for the up and down pressure. The results are presented in Table 1. From Figure 5, it can be deduced that the hysteresis effect was very low and can therefore be neglected. The same occurred with the other sensor cells.

For temperature calibration, all the sensor cells placed in the thermal chamber were subjected to temperature variations ranging from 15 to 40 °C with a 5 °C step, and without any external applied pressure. Although it was verified that the stabilization time of the temperature sensor was around 10 min after the thermal chamber reached the desired value (Figure 4), the response of the FBG_P_ and FBG_T_ was registered 15 min after the temperature stabilization in the climate test chamber in order to ensure more accuracy of the results. The test was carried out for the increase and decrease of the temperature. During this test, the relative humidity of the thermal chamber was stabilized at 70%. 

**Figure 6:**
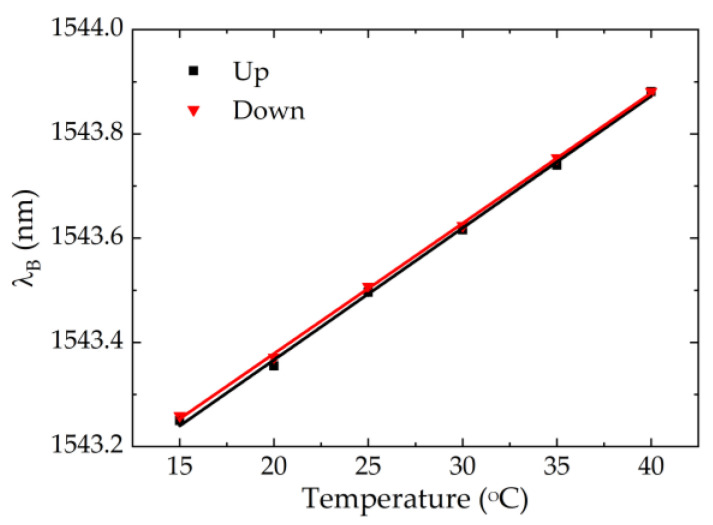
Temperature IL sensor cell calibration test.

Figure 6 shows the results obtained only for the FBG_T_ of the sensor cell IL, both for the temperature increase and decrease. The values were very close, and this trend was verified for all FBG_T_, indicating that the developed sensor cells do not have hysteresis to temperature variations. In terms of sensitivity coefficient of each sensor cell, we considered the mean value of the sensitivity coefficient obtained for the increase and decrease of the temperature (values shown in Table 1).

With the obtained data and respective adjustments and averages, we summarize the main findings in Table 1, wherein the pressure and temperature sensitivity coefficients of FBG_P_ and FBG_T_ are listed for all the sensor cells. The differences of sensitivity coefficients between sensors may have been due to different factors. Although we tried to reproduce the sensor cells in the same way, there were small geometrical discrepancies between sensor cells. Additionally, there were still some steps manually controlled in the process, e.g., placing the resin and gluing and tensioning of the optical fiber. Moreover, the resin can produce small air bubbles that are difficult to control and that can easily change its elasticity and thermal properties, which justifies the differences in the sensitivities observed in Table 1. Concerning the negative values obtained for the FBG_T_ pressure sensitivity coefficient—𝑆_P,FBGT_, this may have been due to a bending of the optical fiber caused by the deformation of the PLA under the applied pressure. Since this bending resulted in a longitudinal compression of the fiber, it will be reflected in a negative wavelength variation. However, the discrepancy in the sensitivity coefficients and the negative wavelength variations achieved in the thermal characterization had no influence on the final results, as the calculations took into account the values present in this table.

### 3.3. Compensation for the Temperature Effect

The pressure and temperature characterization tests were performed, assuming that only one of these parameters varied while the other remained constant. Nonetheless, during the application of the proposed sensing system in the wheelchair, both parameters can vary at the same time. As the FBGs were simultaneously sensitive to temperature and strain under the pressure applied to the sensor cells, it is relevant to compensate the temperature effect on the FBG_P_ response in order to obtain the precise pressure that is applied at each analysis point. For this, a matrix represented by Equation (3) can be considered:

(3) $$\;\left[\begin{array}{c} \Delta \lambda_{{\mathrm{FBG}}_{T}}\\ \Delta \lambda_{{\mathrm{FBG}}_{P}} \end{array}\right]=\left[\begin{array}{cc} S_{P,{\mathrm{FBG}}_{T}} & S_{T,{\mathrm{FBG}}_{T}}\\ S_{P,{\mathrm{FBG}}_{P}} & S_{T,{\mathrm{FBG}}_{P}} \end{array}\right]\left[\begin{array}{c} \Delta P\\ \Delta T \end{array}\right],$$

where $\Delta \lambda_{{\mathrm{FBG}}_{T}}$ and $\Delta \lambda_{{\mathrm{FBG}}_{P}}$ correspond to the Bragg wavelength shift measured in FBG*_T_* and FBG*_P_*, respectively; $S_{P,{\mathrm{FBG}}_{T}}$ and $S_{P,{\mathrm{FBG}}_{P}}$ are the pressure sensitivities of FBG*_T_* and FBG*_P_*, respectively; $S_{T,{\mathrm{FBG}}_{T}}$ and $S_{T,{\mathrm{FBG}}_{P}}$ are the temperature sensitivities of FBG_T_ and FBG_P_, respectively; and Δ*P* and Δ*T* are pressure and temperature variations, respectively. Solving Equation (3) to Δ*P* and Δ*T*, we obtain Equation (4):

(4) $$\;\;\left[\begin{array}{c} \Delta P\\ \Delta T \end{array}\right]=\frac{1}{\det\left(A\right)}\left[\begin{array}{cc} S_{T,{\mathrm{FBG}}_{P}} & -S_{T,{\mathrm{FBG}}_{T}}\\ -S_{P,{\mathrm{FBG}}_{P}} & S_{P,{\mathrm{FBG}}_{T}} \end{array}\right]\left[\begin{array}{c} \Delta \lambda_{{\mathrm{FBG}}_{T}}\\ \Delta \lambda_{{\mathrm{FBG}}_{p}} \end{array}\right],$$

(5) $$\mathrm{with}\;\;A=\left[\begin{array}{cc} S_{P,{\mathrm{FBG}}_{T}} & S_{T,{\mathrm{FBG}}_{T}}\\ S_{P,{\mathrm{FBG}}_{P}} & S_{T,{\mathrm{FBG}}_{P}} \end{array}\right]$$

## 4. Implementation of Sensor Cells in the Wheelchair

**Figure 7:**
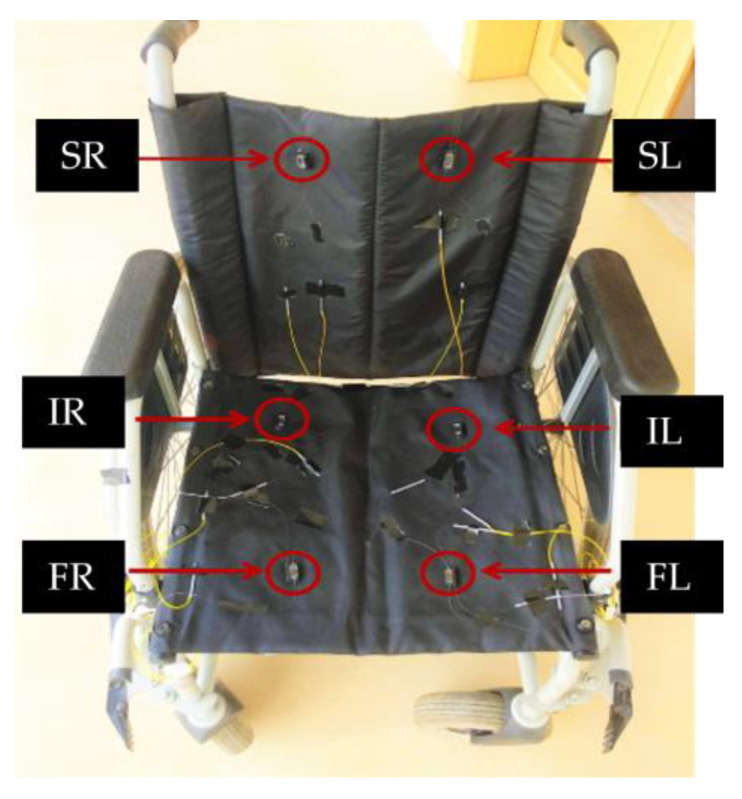
Photography of the wheelchair with the identification of six FBG-based sensor cells for pressure and temperature monitoring.

The sensor cells were attached to the specific areas of interest of a wheelchair, as depicted in Figure 7. Special care was also taken with the optical fiber in order to minimize the curvature, not only to reduce the possibility of breakage, but also to avoid reducing the optical power intensity of the Bragg wavelength peak (due to the optical attenuation caused by the fiber curvature). Although this is not relevant for the measurement itself, as the parameter of interest is encoded in the spectral shift of the Bragg wavelength, this may cause fails on the peak detection by the interrogation system. After the sensor cell network was implemented, foam pads were placed on the back and seat of the chair to protect the sensors and provide more comfort for the user.

**Figure 8:**
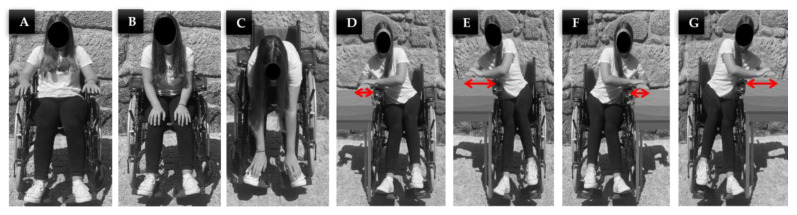
Positions performed during the test: (**A**) reference position, person sitting normally; (**B**) leaning slightly forward with hands on knees; (**C**) leaning strongly forward with hands on feet; (**D**) slight tilt to the right; (**E**) too tilted to the right; (**F**) slight tilt to the left; (**G**) too tilted to the left.

All the tests were performed by the same person, who weighed 58 kg, had no identified pathologies, and was able to move and perform the different positions without external assistance. In accordance with the areas most prone to the development of pressure ulcers, we carried out the protocol demonstrated in Figure 8, consisting of a set of pressure relief positions, as recommended in the literature [7,15]. The tests were realized in a closed indoor environment at a constant temperature.

### 4.1. First Test: Sequence of Pressure Relief Positions

**Figure 9:**
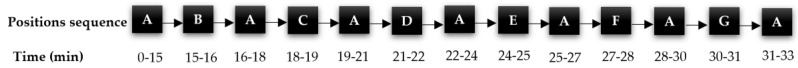
Scheme showing the sequence of pressure relief positions and their time at the first test.

The first test with the instrumented wheelchair aimed at verifying the reliability of the optical sensor network to monitor, in real time, the pressure and temperature in specific areas of the wheelchair during different positions. The test began with the user sitting in position A, during 15 min for temperature and pressure stabilization. The other positions, presented in Figure 8, were performed for 1 min, always interrupted by 2 min in the initial position A for blood flow stabilization. Figure 9 describes the sequence of relief positions during the first test.

Since it is known that the Bragg wavelength increases with pressure, it is possible to predict the FBG_P_ sensor cells response during this test, as described in Table 2.

**Figure 10:**
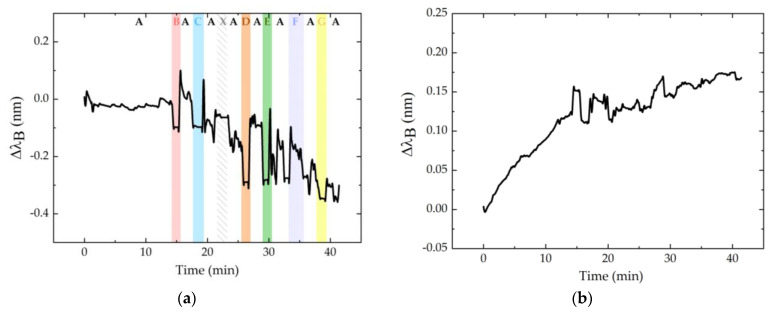
Bragg wavelength shift for the FBG_P_ (**a**) and FBG_T_ (**b**) of the SR sensor cell. In the first graph (**a**), each color refers to the position that was exercised at that moment: position A in white; B in red; C in blue; D in orange; E in green; F in dark blue; and G in yellow. The white area with pattern corresponds to a random movement of the volunteer (identified as X).

Figure 10 shows the Bragg wavelength shift registered for the FBG_P_ (a) and FBG_T_ (b) of the SR sensor cell during the sequence of relief positions identified in Figure 8 and Figure 9.

**Figure 11:**
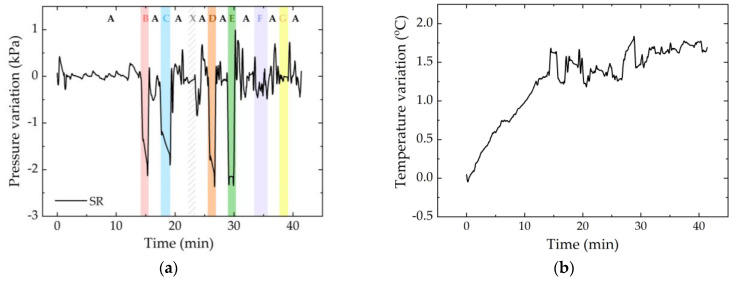
Pressure with temperature compensation (**a**) and temperature (**b**) of the SR sensor cell during the test. In the first graph, each color refers to the position that was exercised at that moment: position A in white; B in red; C in blue; D in orange; E in green; F in dark blue; and G in yellow. The white area with pattern corresponds to a random movement of the volunteer (identified as X).

Using Equation (4) and the sensitivities presented in Table 1, we were able to convert these data to pressure and temperature, represented in Figure 11a,b, respectively. Comparing the Figure 10a and Figure 11a, we found that the difference between the shape of system response before and after temperature compensation, respectively.

**Figure 12:**
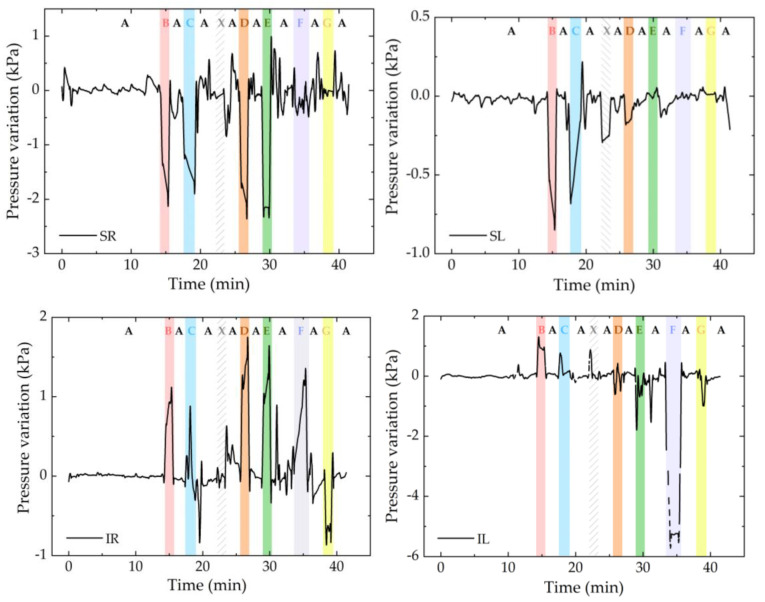

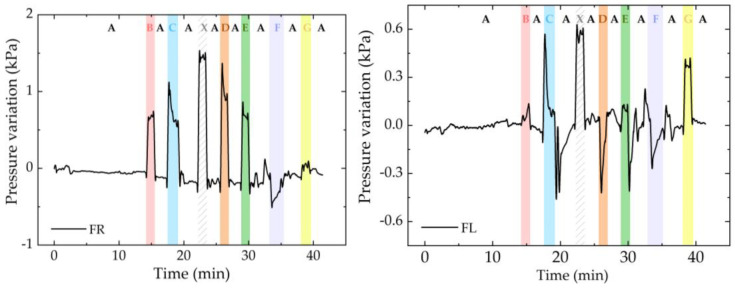
Pressure variation for all sensor cells during the test. In the graphs, each color refers to the position that was exercised at that moment: position A in white; B in red; C in blue; D in orange; E in green; F in dark blue; and G in yellow. The white area with pattern corresponds to a movement of the volunteer (identified as X).

For comparison purposes, Figure 12 shows the pressure variation registered for all the sensor cells.

In general, the response of the sensor cells is similar to the one already predicted (Table 2), although in some cases there were some pressure peaks that affected the values detected immediately afterwards, mainly in position 7. The discrepancy could have been caused by sudden movements that exerted pressure on the FBG_T_ and led to errors in the temperature compensation or even torsion and strain of the optical fibers. However, with time and normalization of the position, these errors can certainly be eliminated.

During the test, the greatest pressure variation, with about 6 kPa, was found in the ischial region (IL), and the greatest thermal variation was also found in the same region (IR), with an increase of 5 °C. This is consistent with the state of the art [7,8], which identifies the ischial tuberosity area as one of the most likely areas for the development of pressure ulcers in wheelchair users.

### 4.2. Second Test: Random of Pressure Relief Positions

**Figure 13:**
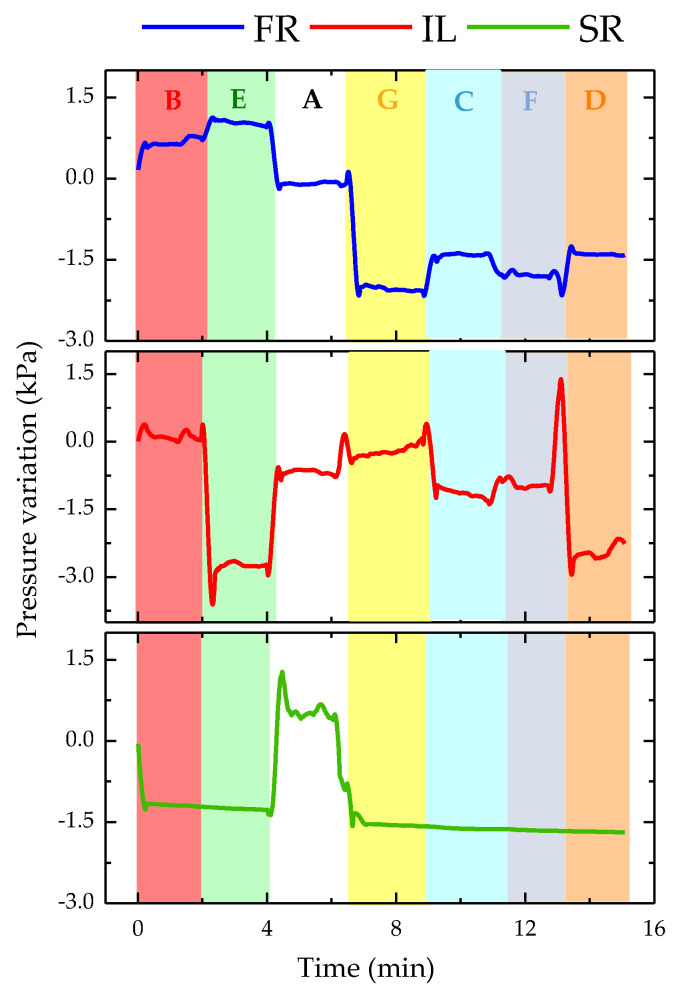
Pressure variation for SR, IL, and FR sensor cells during the random test of relief positions: position A in white; B in red; C in blue; D in orange; E in green; F in dark blue; and G in yellow. The white area with pattern corresponds to a movement of the volunteer (identified as X).

This test aimed to verify the reliability of the proposed optical sensor network for real-time detection of the wheelchair user’s position. For this, the volunteer sat in the wheelchair and randomly adopted the positions described in Figure 8. Since this test was performed immediately after the one described previously in Section 4.1, the initial 15 min required for the temperature stabilization was not considered (this condition was already guaranteed). To identify the seven positions of the wheelchair user, we only needed to analyze the response of three sensors as long as they met two criteria: use of sensors from opposite sides of the seat and use of sensors from all areas of analysis (scapulae, ischium, and femur). Thus, to simplify the analysis, we show in Figure 13 the pressure variation plots only for three sensors, namely, FR, IL, and SR sensor cells.

By observing the data from each of these sensor cells, we were able to deduce the position in which the wheelchair user was in. Table 3 describes the sensor cell behavior and the predicted position, as well as the real position adopted by the volunteer.

Knowing the positions assumed by the wheelchair user, we were able to compare those with the conclusions drawn from the observation of the data presented in Figure 13. In fact, it was possible to deduce the position that the wheelchair user was assuming at that moment from the pressure analysis. However, it should be noted that it was not possible to draw conclusions from the response of only one sensor cell. For unambiguous identification, one must have the response of at least three sensor cells, preferably one from the shoulder blade area and two from the seat area.

## 5. Discussion

Compared with the system proposed by Tavares et al. [15], this work had an improved sensor cell arrangement that allows for the detection of several positions of wheelchair users and a new sensor cell design enabling them to simultaneously monitor temperature and pressure at each point. It was found that the temperature is an important parameter to be monitored, not only because it increases with friction and therefore it is indirectly related to the occurrence of pressure ulcers [14], but also because the FBGs are simultaneously sensitive to both pressure and temperature variations. Therefore, the design of the sensor cell proposed in this work allows for a compensation of the temperature effects on the sensors dedicated to pressure measurements, enabling a precise value of both the temperature and pressure exerted at each point to be obtained.

To validate the performance of the sensor network, we implemented two tests: one consisted of the execution of a well-defined sequence of pressure relief movements (as de-scribed in the literature [7,15]) for real-time monitoring of temperature and pressure; the other test consisted of a random sequence of movements in order to check if it is possible to identify the different positions with the proposed optical sensing network. The pressure variations obtained were within the expected order of magnitude [16]. The sensor network was proven to be reliable, not only for real-time pressure and temperature monitoring, but also for the detection of the wheelchair user position.

On the basis of the proposed sensing system, we were able to perceive the position in which the wheelchair user was in, in order to warn when they are exceeding the recommended pressure values and advise a relief position. However, it is important to note that these values are dependent on factors that are currently not measured, such as the skin’s condition. 

## 6. Conclusions

In this work, a sensing network with six FBG-based sensor cells was developed for simultaneous and real-time pressure and temperature monitoring in a wheelchair. The motivation for monitoring these physical parameters in specific positions of a wheelchair is based on the need for solutions that help in the prevention of pressure ulcer development.

Six specific areas of the wheelchair (four in the seat area and two in the shoulder blade area) were instrumented with the sensor cells. This enabled us to carry out experimental tests in which the response of the sensors to a specific sequence and a random sequence of pressure relief positions was tested. On the basis of the proposed sensing system, we were able to differentiate the response of sensors to pressure and temperature variations, as well as to perceive the position in which the wheelchair user was in, in order to warn when they were exceeding the recommended pressure values and advise a relief position. 

In the future, the development of a low-cost, stand-alone interrogation system installed on the wheelchair can be considered, capable of being powered by a battery. Moreover, regarding the sensor cells production, different casing materials and filling resins could be tested to improve the cell reproducibility and/or performance. Additionally, for the optimization of the sensor cells, it will be important to carry out applicability and usability studies in which a large number of users with and without ulcers can test the system for several hours.

## Figures and Tables

**Table 1:** Pressure (𝑆_P_) and temperature (𝑆_T_) sensitivity coefficients for the FBGs of all sensor cells.

Sensitivity Coefficient	SR Scapula Right	SL Scapula Left	IR Ischium Right	IL Ischium Left	FR Femur Right	FL Femur Left
𝑆*_P,_*_FBGP_ (pm/kPa)	87.0 ± 8.0	46.0 ± 3.0	83.7 ± 0.7	16.0 ± 1.0	89.0 ± 6.0	163.0 ± 11.0
𝑆*_P,_*_FBGT_ (pm/kPa)	−4.5 ± 0.4	−2.7 ± 0.3	−1.5 ± 0.3	−10.0 ± 1.0	−11.4 ± 0.4	−0.2 ± 0.1
𝑆_𝑇__,FBGP_ (pm/°C)	22.0 ± 0.2	54.0 ± 5.0	21.5 ± 0.2	15.0 ± 0.3	18.2 ± 0.1	21.6 ± 0.2
𝑆_𝑇__,FBGT_ (pm/°C)	89.0 ± 1.0	19.6 ± 0.1	27.1 ± 0.7	25.3 ± 0.4	116.0 ± 2.0	9.3 ± 0.1

**Table 2:** Expected variations to be observed for each FBG_P_ of the sensor cells throughout the test.

Sensor Cells	Expected Results
**SR** and **SL**	After the initial position A, in general, there should be a negative variation of the Bragg wavelength in the periods corresponding to positions B, C, D, E, F, and G, since the body is no longer in contact with the sensors when these relief positions are exercised.
**IR** and **FR**	Increased Bragg wavelength shift in the period corresponding to positions B, C, D, and E. Decrease in the periods corresponding to positions F and G, with the latter being more accentuated. In position C, a greater variation on the FR sensor cell than the IR sensor cell is expected.
**IL** and **FL**	Increased Bragg wavelength shift in the periods corresponding to positions B, C, F, and G. Decrease in the positions D and E (more accentuated in this case). In position C, a greater variation on the FL sensor cell than on the IL sensor cell is expected.

**Table 3:** Sensor cells behavior and correspondent position.

Sensor Cell Behavior and Predicted Position	Real Position
In the first 2 min, there was a positive pressure variation in FR and IL, and a decrease in SR, that is, the user did not have the shoulder blades supported on the wheelchair structure and there was pressure in both the femoral and ischial areas (left and right), corresponding only to position B.	B
The pressure in SR was maintained, increased even more in relation to the previous position in FR, and decreased abruptly in IL. This was only verified when the wheelchair user was too inclined to the right, corresponding to position E.	E
There was a positive variation in SR corresponding to the position in which the wheelchair user supported the shoulder blades in the area where the sensor cell was located. This variation occurred exclusively for position A, and was confirmed by the variations observed in the remaining sensor cells.	A
There was a null variation in SR for the rest of the test, that is, position A was not checked again. As there was an abrupt negative variation in FR and a positive variation in IL, it was concluded that the wheelchair user was excessively tilted to the left, being in the G position.	G
The pressure increased in FR and decreased in IL, that is, the wheelchair user was not only exercising pressure on the sensor cells positioned on the left side. As the pressure was higher in the femur area, the user should have been in position C.	C
There was again a pressure decrease in the FR sensor cell and an increase in IL; however, this was not so pronounced, corresponding to position F.	F
There was an increase in the pressure felt by the FR and a decrease in IL; however, both were slight variations, corresponding to position D.	D

## Data Availability

Not applicable.
